# Termination of the leprosy isolation policy in the US and Japan : Science, policy changes, and the garbage can model

**DOI:** 10.1186/1472-698X-5-3

**Published:** 2005-03-16

**Authors:** Hajime Sato, Janet E Frantz

**Affiliations:** 1 Department of Public Health, Graduate School of Medicine, the University of Tokyo, Hongo 7-3-1, Bunkyo-ku, Tokyo 113-0033, Japan; 2 Department of Political Science, the University of Louisiana at Lafayette, Lafayette, LA 70504-1652, USA

## Abstract

**Background:**

In both the US and Japan, the patient isolation policy for leprosy /Hansen's disease (HD) was preserved along with the isolation facilities, long after it had been proven to be scientifically unnecessary. This delayed policy termination caused a deprivation of civil liberties of the involuntarily confined patients, the fostering of social stigmas attached to the disease, and an inefficient use of health resources. This article seeks to elucidate the political process which hindered timely policy changes congruent with scientific advances.

**Methods:**

Examination of historical materials, supplemented by personal interviews. The role that science played in the process of policy making was scrutinized with particular reference to the Garbage Can model.

**Results:**

From the vantage of history, science remained instrumental in all period in the sense that it was not the primary objective for which policy change was discussed or intended, nor was it the principal driving force for policy change. When the argument arose, scientific arguments were employed to justify the patient isolation policy. However, in the early post-WWII period, issues were foregrounded and agendas were set as the inadvertent result of administrative reforms. Subsequently, scientific developments were more or less ignored due to concern about adverse policy outcomes. Finally, in the 1980s and 1990s, scientific arguments were used instrumentally to argue against isolation and for the termination of residential care.

**Conclusion:**

Contrary to public expectations, health policy is not always rational and scientifically justified. In the process of policy making, the role of science can be limited and instrumental. Policy change may require the opening of policy windows, as a result of convergence of the problem, policy, and political streams, by effective exercise of leadership. Scientists and policymakers should be attentive enough to the political context of policies.

## Background

Leprosy, or Hansen's disease (HD), is a chronic infectious disease caused by *Mycobacterium leprae*[1]. The disease has been known since ancient times: The origin of the word leprosy dates back to Greek and Latin[2]. Over a long time period, the disease can be disfiguring, and societies have stigmatized victims of the disease. This attribute is deeply discrediting since the stigmatized individual is disqualified from full social acceptance. Leprosy was thus dreaded, not because it killed, but because it left one alive with no hope[3,4]. After Armauer Hansen's discovery of the bacteria in 1873, the disease became feared as a contagion, and segregation was recommended for prevention. The advent of effective drugs in the 1940s drastically changed the course of disease, and in many countries, patient isolation was deemed unnecessary and terminated. However, some countries that practiced patient isolation experienced difficulties in changing their policies, abolishing leprosaria and reintegrating the patients into the community[5]. As a result, prolonged policies caused the institutionalization of patients for a period longer than scientific knowledge justified.

Public policy termination is often perceived as the final outcome of a political, but highly rational, policy process. When a policy's objectives are reached and maintained, its relevance and applicability should be reconsidered and, if found redundant, outmoded, or dysfunctional, the policy should be terminated[6]. Termination might occur with either a bang or a whimper: public programs and policies may end suddenly, perhaps after a lengthy resistance, or they may end slowly after a long-term decline in the resources with which they are [7,8]sustained. In either case, when termination does not take place properly, the policy may cause harmful effects, either materially or ideologically, rather than beneficial ones[9]. Adopting a policy to terminate a law is always a struggle presenting a number of obstacles, especially. Those posed by agents with vested interests[10]. To overcome various political hurdles, a set of effective political strategies is sometimes required, as well as political maneuvering by those skilled at policy [11-13]termination.

There seems to be a general expectation that the introduction, maintenance and termination of health and medical policies are deliberate, meaning both rational and scientifically justifiable. However, the relationship between science and policy is actually quite complex. It is rarely a linear and rational one in which science finds and establishes the facts and then policymakers incorporate them to solve social problems. This study examines the history of leprosy control policy in the US and Japan, focusing especially on the national legislative changes concerning patient isolation. It seeks to examine the functions of science in policy change, in this case the abolition of the long-standing patient isolation policy. Importance of favorable political context, along with skillful policy entrepreneurs, is then addressed.

## Methods

### Materials

To examine the process of policy making concerning the isolation of patients with leprosy / HD, a comprehensive search and collection of historical documents were conducted both in the US and Japan. After a thorough search of archives of public records, both printed and on-line, using the keywords leprosy and Hansen's disease, relevant documents were exhaustively collected from libraries, leprosaria/sanatoria, offices of patients' organizations, and the museum of Hansen's disease (Carville and Tokyo) in each country. Sources include the Research Library of the National Archives and Record Administration (Washington)[14,15], the libraries at Louisiana State University (Baton Rouge) and Harvard University (Cambridge and Boston), and the National Diet Library (Tokyo). Databases of medical articles, such as MEDLINE[16] and ICHUSHI[17], were utilized to search for publications on leprosy/HD. Other types of documents and books, such as theses, essays and recollections, were also gathered. Supplementary interviews were then conducted during 2000–2004 with leprosaria directors and physicians, administrators, and patients, both past and present, in the US and Japan.

### The Garbage Can model of policy making

The observation of politics involves making observations conform to sets of assumptions, which are called models. These models help delineate the relationships among conditions and patterns in political life. The Garbage Can model of policy making was proposed by Cohen et al[18], and further developed by Kingdon[19] as a contrast to linear, comprehensive, and rational models[20]. This model assumes that policy windows open only when the process streams of problems, policies and politics converge, separately and independent of each other. In such a convergence, problems are brought to the attention of people in and around government by systemic indicators, by focusing events like crisis and disasters, and by feedback on the operation of current programs. Alternate policy proposals are developed from the many possible ideas floated by those both in and out of government. The proposals that survive to achieve serious consideration must meet several criteria, including technical feasibility, a fit with dominant values and the current national mood, budgetary workability and political support. Political support may be affected by a variety of influences including swings of national mood, administrative or legislative turnover, interest group campaigns, and social movements.

In this context, policy windows are the opportunities to push forward one's preferred proposal or conception of the problem. These windows of opportunity in policy making can open either as a result of happenings in the political stream or by the development of compelling problems. In the Garbage Can model, solutions and problems have equal status as separate streams in the system, and the popularity of a given solution at a given point in time often influences which problems are focused on. In other words, collections of choices are looking for problems, issues and feelings are looking for decision situations in which they might be aired, solutions are looking for issues to which they might be the answers, and decision makers are looking for work. Agendas are set by problems or politics and alternatives are generated in the policy stream. So, outcomes are heavily dependent on the coupling of the streams. Sometimes in this process, policy entrepreneurs – people who invest their resources in pushing their pet proposals or problems – are responsible, not only for prompting important people to pay attention, but also for coupling solutions to problems and for setting both problems and solutions in a functional political context.

This Garbage Can model was sometimes employed to explain the dynamics of policy making in the areas other than health care, and proved to be a powerful analytical tool[21]. In this study, the courses of events were analyzed through this model, and important factors for and obstacles to linking science and policy were examined.

With respect to leprosy policy, history after WWII is divided into three periods. Period I covers the time period of the late 1940s through 1950s, when the advent of new drugs could have opened the policy windows in both countries. Related events in Japan seem to have lagged about five years behind those in the US, presumably due to the delayed introduction of those drugs. Period II represents the time in the 1960s and 1970s, when true legislative policy change did not occur, but incremental administrative actions were taken. Finally, Period III encompasses the 1980s and 1990s, when the policy of patient isolation was legislatively abolished in both the US and Japan. Relevant facts preceding Period I are also succinctly presented.

## Results

### Isolation of leprosy patients in the United States

#### US background

In 1865, Hawaii established a policy for the quarantine and isolation of HD patients. A leper settlement was established at Kalaupapa on Molokai island, and the Kalihi Receiving Station was opened on Honolulu to diagnose cases and provide emergency treatment[22]. In 1873, when *Mycobacterium leprae *was discovered by Hansen and leprosy was thus proved to be a contagion, the seclusion of patients in their homes or in hospitals was advocated for disease prevention[23]. By 1894, Louisiana had established a leprosarium, and several other states, such as New York and California, had also introduced similar isolation policies for HD patients[24]. The First International Congress on Leprosy held in Berlin in 1897 recommended isolation as the appropriate policy measure against leprosy. In 1909, the U.S. government built another leper colony in the Philippines on the Island of Culion.

After several national and state surveys of HD patients, the need for a national policy to control HD and the desirability of patient isolation were discussed repeatedly during the 1890s. The first legislation, passed in 1898, authorized a thorough investigation of leprosy. A commission was appointed, and based on its recommendation, legislation was introduced that allowed for the establishment of a national leprosarium for the segregation of lepers with the goal of preventing the spread of leprosy in the United States[25]. It is worth noting that scientists were never unanimous about the necessity of isolation. Scientific publications at the time described HD as a relatively non-contagious disease[26].

After succeeding reports and debates, legislation was passed in 1917 that provided for purchase of a site and receipt of any person afflicted with leprosy who presented himself or herself for care, detention, and treatment, or who might be apprehended under authority of the United States Quarantine Acts[27]. The American Academy of Medicine, the American Medical Association, and the American Dermatological Association expressed their support for the isolation policy, arguing that patient isolation serves for disease control and patient treatment, as well as protection of patients from social stigma and ostracism. The 1917 legislation also directed that public health employees assigned to the institution be paid one and one-half times the regular pay due to the disagreeable and dangerous nature of the service[28].

Having failed to locate a new site, in 1921 the U.S. government took over the Louisiana Leper Home in Carville, Louisiana, and the United States Public Health Service (PHS) opened the US Marine Hospital Number 66 as a National Leprosarium[29]. The rules "Regulations governing the care of lepers: regulations for the government of leprosaria and for the apprehension, detention, treatment, and release of lepers" were drawn up by the Surgeon General in 1922 to implement the 1917 legislation. The 1922 rules reiterated the admission options (voluntary or involuntary), prohibited patients from leaving the sanitarium grounds, and provided that patients shall not "hold communication" with patients of the opposite sex. The rules also outlined a procedure for discharge of patients, which included a period of one year of special observation for any signs of leprotic retrogression after medical tests showed no signs of the disease. After this year, the patients were requested to appear before a board of three or more medical officers who might pronounce the patient to be no longer a menace to public health[30].

The institution was gradually expanded in the year 1933, having 65 rooms and outpatient facilities in 1941. By 1945, there were 369 inpatients[31]. During those years, there was no really effective treatment of the disease. Most of those at the facility were involuntarily admitted (only 15% of admissions between 1930 and 1945 were reportedly voluntary)[32].

#### US period I (1940 through 1950))

In 1941, Dr. Guy Faget at the National Leprosarium started a clinical trial of Promin, a sulfone drug, for the treatment of HD. Its beneficial effects became rapidly known and were reported within a few years in leading medical journals, such as Public Health Reports and Journal of the American Medical Association (JAMA)[33]. Other forms of sulfone drugs, Diasone and Promazole, were also developed and replaced the traditional HD treatment of applying chaulmoogra oil[34]. The international academic community paid close attention, and the 1946 Conference on Leprosy in Rio de Janeiro reported significant advances from the introduction of the sulfone group of drugs[35].

The enactment of the Public Health Services Act of 1944[36] could have provided a forum for discussion and revision of the US HD policy. Parts D and G of the Act, however, literally duplicated the 1917 Act, apparently without discussion. As before, its Section 331 states – "that the Service (PHS) shall, in accordance with regulations, receive into any hospital of the Service suitable for his accommodation any person afflicted with leprosy who presents himself for care, detention, or treatment, or who may be apprehended under section 332 (regulation) or 361 (quarantine) of this Act, and any person afflicted with leprosy duly consigned to the care of the Service by the proper health authority of any State, Territory, or the District of Columbia. The Surgeon General is authorized, upon the request of any health authority, to send for any person within the jurisdiction of such authority who is afflicted with leprosy and to convey such person to the appropriate hospital for detention and treatment."

Only a year later, in 1945, the American Public Health Association advised against isolating people with leprosy. It was by then well established that the majority of the population had a natural immunity to leprosy, and that it was only mildly contagious to the rest. Since the relative infectiousness of the two types of leprosy had been measured by epidemiological studies, which demonstrated the very low infectiousness of the tuberculoid type, experts insisted that only open (infectious) cases, if any, required isolation[37]. However, at that point, scientists were again not unanimous concerning patient isolation policy. Dr. Faget, who should have known the effects of sulfones better than anyone else, wrote in Public Health Reports that the only reliable means to eradicate leprosy is isolation. Dr. G. W. McCoy, an ex-official of the PHS and Dean of the Louisiana State University Medical School, on the other hand, recommended that since infectivity varies depending on clinical types, the universal isolation policy should be abandoned. He stressed that when there is room to question infectiousness, it should be used to serve patients (meaning to protect patients' liberty)[38].

In response to these debates, the Federal Security Agency (FSA) set up under the Surgeon General the National Advisory Council on Leprosy. The Council discussed the issues raised by the United Patients' Committee for Social Improvement and Rehabilitation – composed of the Patients' Federation, Veterans/Legionnaires, and the staff of *The Star *(a monthly periodical published by the HD patients) – which had expressed its objection to the Public Health Services Act. Abolition of forced isolation and detention and the broadening of outpatient treatment services were discussed, but only the latter was supported[39]. After a while, an experimental project for outpatient services was established in New Orleans.

Efforts to revise the law began soon after the enactment of Public Health Services Act. In 1948, G. H. Rarey (US Army – retired Colonel, national vice president of the American Federation of the Physically Handicapped) and Paul Strachan (president, American Federation for the Disabled) drafted the National Leprosy Bill, and asked Congressman Charles J. Kersten (R-Wisconsin) to submit it to Congress[40]. In the following year, a similar bill, the National Leprosy Act was submitted by Senator Claude D. Pepper. Both of these draft legislation intended drastic changes in leprosy control policy, including: dissemination of pertinent facts concerning leprosy (to promote an enlightened public opinion, a new and more accurate understanding of leprosy); treatment of leprosy patients (to establish leprosy treatment principles, methods, and regulations for administering leprosy treatment more in harmony with the customs of society as applied to the care and treatment of persons afflicted with other diseases; to arrange treatment centers, both inpatient and outpatient services); the National Advisory Council on Leprosy; rehabilitation and reemployment of leprosy patients; financial assistance for leprosy patients and their dependents; compensation for disability incident to leprosy; and expansion of leprosy research.

At the Congressional hearings on the bills, the experts' testimony took different positions[41]. As a drafter of the bill, Colonel Rarey, who had served in the Philippine Islands and encountered many leprosy patients, argued insistently against the segregation of leprosy patients, claiming that it was not effective, could destroy patients' social lives, and might hinder patients from seeking proper treatment. He stated, "Many cases of this disease are not communicable. The remainders are classified by leprologists as feebly communicable. The status and low degree of communicability of leprosy in the US does not justify continuance of the present compulsory segregation policies." He also criticized the PHS, arguing that the FSA's favoring of segregation erred on the ultraconservative side of this question.

On the other side, J. Donald Kingsley, Acting Administrator of the FSA, presented opposition to the bill. Although he indicated sympathy with the basic objectives of the bill, the necessity for law revision was denied. He argued that most of the objectives of the bill could be more effectively achieved through intensification of activities already authorized by the existing law. The bill was criticized as defective in attempting to divide responsibility for the treatment and care of patients not hospitalized in PHS hospitals, between the PHS and the various federal, state, and local governmental agencies. Especially stressed was the point that the proposed Act does not meet squarely the problems of forced detention of leprosy patients: It was insisted that the existing authority of the PHS should not be repealed or cast in doubt without careful consideration and provision for alternative methods of meeting the problem, should need arise. The FSA, while recognizing the accumulating evidence showing the effectiveness of the sulfone drugs, thus retained a conservative position.

The arguments of the FSA were accepted, and as a consequence, those bills, though submitted repeatedly through 1954, were never enacted. The only leprosy-related legislation passed in this period was a bill enacted in 1951, which dealt with transportation costs for released patients[42,43]. At the administrative level, however, a series of changes was introduced. In 1946, patients were given the right to vote. In 1947, the PHS removed HD from the list of quarantinable diseases which required a travel permit. In 1948, the barbed wire fence was removed from the facility, and the first active patient was released to the care of her private physician. In the same year, the PHS officially recognized the name Hansen's disease as a replacement for the term leprosy. In the 1950s, patients were allowed to marry. Twelve negative monthly tests were no longer required for discharge; instead those who qualified for discharge were required to be financially stable and assure authorities that there were no children living where they intended to go[44].

#### US period II (1950 until 1980)

In the early 1950s, patient isolation was criticized more and more and eventually condemned at the International Congress on Leprosy and at other international conventions [45]. Following advice that the isolation of patients should be limited to infectious cases, the abolition of compulsory isolation was repeatedly recommended also by WHO[46] and UNICEF[47]. Definition of the cases suitable for temporary isolation gradually became delineated[48].

In 1952, a decision was made that no administrator should stay at the institution for more than three years. Accordingly in 1953, the government appointed a new director, Dr. Edward M. Gordon, a former director of the PHS hospital in Chicago. Soon after his arrival, he announced that those who did not need to be medically hospitalized should leave the institution[49]. His policy was that those able-bodied residents diagnosed as arrested should be discharged; that discharge should be recommended to those who were arrested and partially disabled; that those who were disabled (blind or physically handicapped) could stay in the institution, but could leave if their relatives or friends offered appropriate care. He also passed a rule prohibiting able-bodied residents from working in the institution. Fearful of losing their homes and jobs, some of the patients sent lawyers to Washington to lobby elected officials[50]. After three years, Dr. Gordon left for Fort Monroe in Virginia, and Dr. Edgar B. Johnwick was appointed as the new director. In 1956, on the third day of his duty, Dr. Johnwick declared before the patients that no one would be discharged from the hospital against his/her will and that no one would be kept in the hospital against his/her will[51].

During this period, scientists, while acknowledging the clinical effects of sulfones and the distinction between infectious types, were not unanimous in negating the utility of patient isolation. Dr. L. F. Badger of Leprosy Control Unit, the Communicable Disease Center (later, renamed as Center for Disease Control), stressed in 1956 the importance of isolation of infectious patients, as well as the need for patient followup and continued treatment of arrested cases[52]. Conversely, in 1958, Dr. Meyer, Medical Director at Carville, insisted that as disease control measure, isolation is not necessary in many cases and has serious defects[53]. No overt controversies were found after that in the US literature. Meanwhile in 1960, the World Health Organization advised against isolation and in 1963 at the 8th International Leprosy Congress in Rio de Janeiro, the International Leprosy Association recommended against isolation and furthermore recommended that leprosy needed no special care beyond that afforded other communicable diseases[54].

The last compulsory isolation was reportedly enforced in 1960, and Part 32 of the Federal Code of Regulations "Medical Care for Persons with Hansen's Disease and Other Persons in Emergencies" dropped the term "detention" in 1975[55]. It should be noted that all these policy changes were made through administrative action, not by changing statutory law. Law revision was not visible on the agenda as these policy changes proceeded. Even in 1974 when Hawaii ruled that all future new cases of the disease would be treated as outpatients, no debate took place on the federal level[56]. Compulsory isolation was terminated as the practice of involuntary admission and that of forced institutionalization ended.

#### US period III (Post 1980 through 2000)

The driving force to terminate the isolation policy legislatively and abolish the leprosarium came not from new medical findings but from arguments for economic efficiency and budget cutting. In 1981, the Reagan administration and Congress agreed that it no longer made sense for the government to run public health hospitals, since Medicare, Medicaid, and the general oversupply of hospitals made specialized hospitals obsolete. A congressional subcommittee, backed by the Omnibus Budget and Reconciliation Act, thus targeted the nation's nine public health hospitals, including the National Leprosarium at Carville[57]. However, lobbied by the several groups which had developed over the years to oppose termination – including the community of Carville, the patients, a society of Legionnaires and veterans -, Congressman Gillis Long fought for an exemption for Carville. Consequently, the leprosarium survived the challenge based on the argument that HD patients need special care and considerations (The institution was renamed as Gillis Long Hansen's Disease Center later in 1986)[58].

To the Congress, after 1981, Congressmen Henry A. Waxman and Gillis W. Long instead proposed a bill to change HD policy. Avoiding the issue of closing down the Carville institution, the bill was intended to permit treatment of HD outside PHS facilities and to eliminate additional pay for personnel treating HD. In the report attached to the bill[59], Richard Ashbaugh (Acting Director, Bureau of Medical Services, PHS) indicated the PHS's support for the bill. He stated, "Leprosy pay ... was instituted because of a mistaken belief that workers were at significant risk in contracting Hansen's disease ... Not only is leprosy pay an unjustifiable expense, it actually increases the stigma associated with Hansen's disease." He went on to say, "The PHS is strongly committed to the least restrictive possible treatment of Hansen's disease ... " A similar act was submitted in the subsequent year, but also not enacted at least in part because beneficiaries of the center's payroll, including the patients working at the facility, made a strong objection to the bill.

Without legislative support, the PHS had been subsidizing ambulatory care centers for HD patients, outside the HD Center since 1981. The PHS National Outreach Program started 11 regional outpatient services nationwide[60]. When the PHS in 1983 did a formal review of the Center, it issued a report which highlighted the Center's economic inefficiency, making that the focal point of the discussion. The Center at that time housed only 200 patients but comprised 98 buildings on 337 acres. The staff included 317 civil service and PHS workers and 125 part-time patient employees. The PHS recommended that the Center continues its custodial care and research programs, but asked for more outpatient clinics and a study of the elimination of residential care. In the following year, the PHS set up a utilization review committee in Carville to review periodically the conditions of patients who had been hospitalized more than two years, for their possible discharge ability[61].

Finally, legislation was made. In 1985, PL99-117 was enacted which stated the PHS ... "shall provide care and treatment (including outpatient care) without charge ... to any person suffering from Hansen's Disease who needs and requests care and treatment for that disease"[62,63]. The term "detention" was deleted from the Act, though the provision of residential care was not completely negated. The hazardous duty salary supplements, while had been reduced to one and one-quarter time, were discontinued for new hires.

In 1988, three years after the legislation, the PHS published its report "Strategic Plan, National Hansen's Disease Center (NHDC)[64]." The plan reiterated the situation of the NHDC, and evaluated whether it would be cost effective and feasible to contract out the patient care activities of the Center and transfer the research activities elsewhere. The report recommended expanding the Center's mission to include other nerve-desensitizing diseases, contracting out long-term patient care, and moving the Center's research facilities to Baton Rouge. It also argued the necessity of maintaining the Center so that patients who had lived at Carville for decades could be cared for compassionately in familiar surroundings. The report reached the definitive conclusion that there should be no new resident admitted to the Center, so that over time the population would dwindle to nothing and the entire facility could be closed. Accordingly in that same year, Dr. John Duffy, a new director, began the move of the Center's acute care, research and educational functions to Baton Rouge, while allowing the current residents to stay in Carville[65]. Thus, in practice, both voluntary and involuntary isolation and hospitalization ended.

Nonetheless, despite these developments, closure of the leprosarium required more than a decade, thwarting both a 1990 plan to transform the institution into a geriatric prison and a 1996 plan to make it a federal prison for minimum security geriatric patients. The efficiency argument made by the PHS was always counterposed by the equity and civil rights arguments offered by the patients and their allies. The patients, though demanding that their basic human rights and freedom be restored, claimed a right to lifelong care by the government[66]. Only the research branch was moved in 1992 to a location at Louisiana State University in Baton Rouge.

Then, agenda was set again by the economic efficiency argument, but this time with a new set of policy alternatives. Representative Richard H. Baker proposed that the site be used as a job training school, and that patients be given an annual living subsidy if they departed[67]. Baker, US District Judge Frank Polozola, and Jim Mitchell (PHS) visited Carville to discuss with patients and staff members the idea of closing the Center. This proposal was quashed once when Congressman Cleo Field, who represented the district from 1992 to 1996, objected. However, when the 1996 election returned the district of the Center (District 6 of Louisiana) to Baker, he resubmitted the bill with the proposal that the site be transferred to the State of Louisiana[68]. This time his plan was adopted.

PL 105-78, "Relocation of Gillis W. Long Hansen's Disease Center" was signed into law in 1997[69]. The legislation returned the physical facility to the State of Louisiana without charge, though it specified that the Carville site must be used for health or educational purposes for 30 years. It offered a voluntary separation incentive payment to civil service employees. It directed that at or through the Center, the Secretary of the Department of Health and Human Services (DHHS) must provide short-term care and treatment without charge, including outpatient care, for Hansen's disease and related complications. The Secretary, however, was not permitted to provide long-term care for any disease or complication through the Center. For long-term-care patients, the Secretary was instructed to provide for long-term care without charge for the remainder of the life of the patient. The bill also directed the relocation of the patients at the Center to Baton Rouge within three years unless such relocation was not feasible, and it reassured the remainder that they could stay at Carville as long as they were able to live independently. It further offered a $33,000 annual stipend to any patient who chose to leave the institution, and this irrevocable option could be chosen at anytime by a patient. In August 1999, the federal government transferred the leprosarium to the State of Louisiana for use as a Job Corps training site. The Louisiana National Guard initiated its Youth Challenge program shortly after that[70]. Some patients left Carville going either to the Baton Rouge facility or to the other places, but quite a few remained. Today, the NHDC in Baton Rouge admits about 180 HD patients, and treats several hundred patients annually on an outpatient basis[71].

### Isolation of leprosy patients in Japan

#### Japan background

Since ancient times, historical records indicate, many HD patients lacked permanent homes, leaving them to wander around living in both towns and rural areas, and sometimes creating their own colonies. Leprosy had been understood to be a hereditary disease, but since the mid 19th century, the view that it was the result of a contagion became known gradually, first among the medical experts, and then among the public[72]. The recommendation of the first International Conference on Leprosy held in Berlin in 1897 led Japanese experts to support patient segregation, although not always unanimously. A national survey was conducted about leprosy, and Law No. 11, "The Act on Leprosy Prevention," was passed in 1907. At that time, the Ministry of Internal Affairs (MIA) explained that despite the mild infectiousness of leprosy, Law No. 11 was required for the sake of vagrant lepers, those without means of support[73]. Five public leprosaria were established by local governments in 1909. Voluntary organizations and local governments launched the No Leprosy Movement in 1924, which tried to find all leprosy patients and send them to the leprosaria.

Subsequently, in 1931, the first national leprosarium was opened, and at that time, Law No. 11 was revised to the Leprosy Prevention Law, which allowed all patients, whether they could be cared for at home or not, to be hospitalized without any financial burden levied on their families[74]. The Ministry of Health and Welfare (MHW), the successor of the MIA, nationalized all of the existing leprosaria so as to coordinate their activities. Ministerial officials issued manifests that leprosy should be eradicated from Japan through absolute segregation[75]. In accordance with the increasing institutional capacity of leprosaria, the number of patients sent to leprosaria increased considerably.

#### Japan period I (1940 through 1950s)

The sulfone drug Promin came to Japan in 1946 shortly after WWII ended. Its significantly beneficial effects were reported shortly at the Congress of the Japan Leprosy Association[76]. However, many physicians remained unconvinced of the real efficacy of the drug: They argued that the disease could relapse, even though it might show initial response[77,78]. The new drugs were difficult to obtain either by import or by domestic production, but patients were desperate to try them. They organized the Federation of National Leprosarium Patients (FNLP) and launched lobbying activities to acquire the drug. In response, the Diet (the legislative body in Japan) approved in 1950 a budget for sulfones. The drugs were delivered to leprosaria, and, a year later, the first 35 patients were officially discharged as a result of their improvement[79].

Meanwhile after WWII, during the late 1940s and early 1950s, a new government was established in Japan, and legal systems were thoroughly reviewed and revised in compliance with the new Constitution. As part of that process, leprosy wound up on the agenda in the Diet in the early 1950s during discussions of social security systems. Debate went on until the revision of the existing law of 1931.

On February 15, 1950, leprosarium directors Drs. Kensuke Mitsuda, Yoshinobu Hayashi, and Ryoichi Yajima testified at the Health and Welfare Committee of the House of Representatives, along with the Director of the Medical Affairs Bureau, the MHW[80]. They insisted on the necessity of continued isolation of leprosy patients, argued for the desirability of the government subsidy for Promin, and recommended the expansion of leprosaria. On October 10, 1951, the Health and Welfare Committee of the House of Councilors heard testimony from five experts, including three leprosarium directors, Drs. Hayashi (then also President of the Japan Leprosy Association), Mitsuda and Miyazaki. Dr. Hayashi stressed the fact that there were approximately 9000 institutionalized patients, and 6000 outside the leprosaria who posed a threat of infection. He argued that leprosaria should be expanded to institutionalize all of the latter and that hazardous pay (about 5–10% at that time) should be increased as in the US. Dr. Mitsuda suggested that a more stringent law should be implemented to require isolation in order to prevent spread of the disease. He contended that though the necessity of isolation is sometimes refuted in the US, leprosy, regardless of its types, must be isolated to prevent infection and that sterilization should be recommended to patients for this purpose. He stressed that the new drugs, although promising in treatment, could not be expected to eradicate leprosy. Dr. Miyazaki essentially agreed with Dr. Mitsuda, adding that patients, many of them with deformities and disabilities, could not be released from leprosaria while they remained unaccepted by society and unable to live outside. He suggested that public enlightenment efforts would be advisable. The other experts argued for expansion of research into leprosy[81].

The bill to revise the Leprosy Prevention Law was submitted as a cabinet bill in 1953. The Minister of Health and Welfare explained to the House members that leprosy is hard to cure, that patient isolation is the only measure that prevents leprosy infection, and that the welfare of patients and their families should be protected by the government. Masayoshi Yamaguchi, Director of Public Health Bureau, the MHW, stated that patient isolation is the sole measure for leprosy prevention and protection of the public welfare, and that compulsory isolation should be warranted by law as a last resort. He suggested that patients might be discharged when leprosarium directors consider their isolation unnecessary and recommended that temporary leave also be allowed at the discretion of directors[82]. Facing the possibility of more stringent laws, the Patients' Federation expressed concern that the prospective law under consideration was focused too narrowly on social protection, while ignoring patients' rights[83].

The revised Leprosy Prevention Law of 1953 was in essence a reflection of the arguments of the experts and maintained a legal basis for compulsory isolation of patients proven to have bacilli and for prohibition of leave without permission. In response to patients' pleas, nine supplementary resolutions were adopted, including provisions for living stipends and patient work, improvement in living conditions, promotion of research, and installation of rehabilitation facilities. The MHW did initiate programs for patient rehabilitation, but the Ministry was still devising a plan to expand leprosaria capacity to hospitalize leprosy patients. The Notice of the Vice-Minister, issued soon after the promulgation of the Law, still described isolation as the only reliable means of prevention of the spread of the disease. A tentative standard for discharge was officially detailed by the MHW in 1956, but it was stated as not being intended to facilitate discharge[84]. In practice, about 500 patients were admitted or readmitted to leprosaria in 1956, while fewer than 100 were discharged.

#### Japan period II (1960s through 1980s)

In 1961, the legislature of the government of the Ryukyu Islands, then under the auspices of the US army, took a major step when it passed the Hansen's Disease Prevention Act. It provided that the Chief Executive may advise hospitalization and may also order an improved patient to leave the hospital[85]. Inspired by the policies of the Ryukyu Islands and acquainted with the international recommendation that most leprosy patients should be treated on an outpatient basis, thus abolishing compulsory isolation, the Patients' Federation (FNLP) repeatedly voiced support for revision of the Leprosy Prevention Law of 1953[86]. They repeatedly insisted that the Law lacked a scientific basis and violated human rights, and argued that they were victims of enforced segregation and of social stigmas fostered by the Law[87]. In 1963, the Patients' Federation filed its petition for the revision of the Law, sending more than 200 patients to pressure the Diet and the MHW.

Despite these efforts, the Diet did not add revision of the Leprosy Prevention Law to its agenda. The MHW officials argued that the existing law legitimized compulsory isolation of leprosy patients, which in turn constituted the legal basis for the government's responsibility to provide them with care and a comfortable living environment. Revision of the Law might eliminate the leprosaria, making it impossible for the patients to live on public support. Overt transition of leprosy control to an outpatient basis could also lead to a similar outcome. It was also argued that the transformation of leprosaria from medical facilities to rehabilitation facilities would be difficult because of budget constraints[88]. Concerned about the future of leprosaria, patients did not have the unanimity necessary for law revision. A 1965 survey disclosed that only 16% of patients had an intention to be discharged in the future while the rest felt that they were unable or unwilling to leave their leprosaria[89].

Leprosarium directors in Japan gradually relaxed the implementation of the law. Patients became increasingly free to leave their leprosaria, and abscondments were not punished after the 1960s. In addition, treatment began to be offered outside leprosaria, although on an informal outpatient basis[90]. Concerned that law revision could eventually harm patients' welfare, patients, as well as the officials in charge, came to pay more attention to improving the leprosaria and providing other benefits for patients. The preservation of the Law was implicitly justified as a way to guarantee patients' welfare.

In May 1972, the Ryukyu Islands, where leprosy treatment was based on outpatient services, were returned to Japan. This return again focused the attention of both patients and medical professionals on policy issues. In 1976, when the Federation of Leprosarium Directors drafted a revision proposal which incorporated specific discharge codes, and then submitted it to patients for their consideration, the Patients' Federation objected to the proposal based on the concern that the adoption of specific discharge codes could result in forced discharge[91]. As was seen in the 1960s, many patients remained hesitant to address the issue of law revision, and their movements focused more on compensation for low-wage patient labor and redressing the long-term compulsory segregation of the past. A decade later, in 1984, the Patients' Federation itself created a committee to examine revision of the Law, but continued to be concerned about the fate of their adopted homes, the leprosaria[92]. Consequently, the Federation of Leprosarium Directors only directed that leprosaria should not be abruptly discontinued as many of their residents were already too old to leave.

The MHW endeavored to improve living conditions in national leprosaria in response to repeated pleas filed by patients, as well as discussions in the Diet. Medical staffing was increased, patient labor was gradually reduced or made more rewarding, and government allowances were increased for leprosaria and their patients[93]. Officials did warn patients of the possibility that the numbers and sizes of leprosaria could be reduced as part of administrative reform if revisions to the Law were implemented too quickly.

#### Japan period III (1980s through 2000)

When Fujio Otani became the Director General of the Tofu Kyokai Foundation, which had been established to serve patients with leprosy, the stalemate in policy change began to resolve. Otani was a medical officer in the MHW, and had been Chief of the Section of National Hospitals and Sanatoria in the 1970s, and Director General of Medical Affairs Bureau in the early 1980s. Recruited to the Foundation after his retirement, he also served as the Chairman of the Advisory Council on Public Health in the MHW. Since the recommendation in the mid 1980s of the UN Commission on Human Rights to improve psychiatric treatment in Japan, Otani had been inspired to act for the protection and restoration of human rights among patients[94]. In 1989, when the Patients' Federation consulted with him on its draft petition on the law, he began to commit himself to the issue of law revision.

In 1992, encouraged by Otani, the Patients' Federation filed a petition with the Minister of Health and Welfare. Although many patients still worried about the fate of their leprosaria, Otani promised them that their homes would be maintained[95]. In the following year, the Tofu Kyokai Foundation was officially consulted by the Ministry on the prevention policy for leprosy. A committee was established, composed of leprologists, medical experts, lawyers, media representatives, bureau officials and patients, and presided over by Otani. He tried to appeal to public opinion by establishing the Museum of Hansen's Disease (MHD) and hosting a series of symposia about the policies on HD[96]. In 1994 at the 67th Congress of the Japan Leprosy Association (JLA), Otani gave a special lecture and stated that the existing Leprosy Prevention Law should be abolished since it was not scientifically justifiable and violated patients' human rights. Six months later, as a result, the Federation of Leprosarium Directors and the JLA publicly confirmed his opinion that the existing law should be abolished[97].

Upon the release of the report by the MHW committee in 1995, the MHW organized an internal panel of experts on the abolishment of the Leprosy Prevention Law. This panel, again chaired by Otani, subsequently submitted its report which recommended abolition of the Law, continued provision of public support for existing patients, and the use of the term "Hansen's disease" in place of "leprosy" in laws and regulations. Finally in 1996, the "Act to Abolish the Leprosy Prevention Law," drafted by the MHW, was passed. It abolished the 1953 Law and at the same time codified the government's responsibility to provide existing HD patients with continuous medical and other social services. It was also provided that patients could either leave the leprosaria or stay there as long as they wished, and that those who decided to come back to the leprosaria after their discharge could be readmitted.

At the time of the abolition of the old law, there were 5,413 patients in leprosaria, whose average length of stay was more than 40 years and whose average age was 72 years old. Only six of these patients actually left their leprosaria in the following two years[98]. In response to lawsuits filed in the late 1990s, in 2001 the court awarded financial settlements to those who had been isolated under the earlier laws[99]. The awards amounted to between $65,000 and $114,000 per person depending on the degree of suffering. Additional pay to the workers at leprosaria was preserved.

## Discussion

This study demonstrates that the isolation of leprosy patients was introduced and made rigorous in both countries around the time when it became known that the disease was contagious. Patient isolation policies and leprosaria were maintained long after it became known that isolation is not necessary in the majority of cases. Remarkable was the stagnation of policy change in the post-war period. The preservation of the isolation policy provided patients with some social support, but continuously deprived them of their civil liberties. Furthermore, the policy as an authoritative statement on the disease may have fostered the social stigma associated with a belief that the disease is a dreadful contagion, thereby maintaining a hurdle to patients' reintegration into society. Evidently, the policy's abolition was not easily accomplished nor was achieved solely by advances in scientific knowledge.

### Garbage Can model of policy making

Kingdon's Garbage Can model is very useful in understanding the failure of 'appropriate' policy termination, as well as its delayed termination, observed in both countries: Any policy has an inertia and efforts to abolish the patient isolation policy did not bear fruit until three streams, i.e., problem, policy, and politics, converged after the mid-1980s. Science, although presumably a potent factor, was not in itself a major driving force in opening a policy window.

In the early post-WWII period (late 1940s through 1950s) both in the US and Japan, scientific developments such as learning that the infectiousness of leprosy is usually feeble and uneven across disease types and the development of sulfone drugs effective against the disease might have been expected to be the major factor in reconsideration of the traditional isolation and institutional policy for leprosy management. In reality, however, the laws were revised, not as a result of the scientific developments, but as a spillover from other policy agendas: the enactment of the Public Health Services Act in the US, and the revision of social security systems in Japan, without substantial policy changes. Once legislated, they could hardly be challenged.

Most actors then participating in the policy discussion were well aware of the new drugs, and patients were anxious to reap the fruits of these drugs and achieve the restoration of their civil liberties, sometimes participating in political mobilization for law revision. Scientific and medical advances could have altered the face of issues in the problem stream, helped generate alternative methods of disease management in the policy stream, and changed the opinion of the public and experts in the political stream. However, many influential experts, who had been engaged in the establishment and/or expansion of leprosaria were determined to keep the policy and the institutions and played on remaining public fear of leprosy[100]. Those conservative "elites", primarily bureaucratic agency representatives in the US and medical professionals in Japan, manifested opposition to the proposed law revision, and their arguments were not critically reviewed and more or less accepted by the other actors. Consequently, science and medicine were unable to play a major role in changing the political environment. For these reasons, a valuable opportunity for legislative change was lost in this period when patients were still young and had not become so dependent on their sanatoria in both countries.

After the unsuccessful exploitation of these incomplete policy windows, the three streams of problems, policies and politics did not converge for several decades. By the late 1960s, it could be said that a near consensus had developed in the research community that isolation was an unreasonable solution. Moreover, the infringement of civil liberties was perceived to be a problem, a perception which increased in importance as academics and international health communities repeatedly recommended the abolition of isolation policy as scientifically unjustifiable.

In the problem stream, focusing events were created by patients, administrators and medical professionals with the goal of encouraging the public and politicians to put the issue on the agenda. Most obviously, patients published periodicals, invited media persons to their meetings, petitioned bureaucracies, and lobbied legislators in both countries. In Japan, patients even conducted sit-ins around the Diet and the Ministerial buildings. The repeated recommendations of international organizations, and the occasional official reports recommending outsourcing and outpatient services, also served as focuses. In the political stream, there were some changes as well. As the public's fear of leprosy dwindled (or at least became latent as they forget about the disease), so did the associated stigma. Improved public attitudes toward the disease meant that the political climate was more favorable to returning the patients to society, terminating the isolation policy, and abolishing leprosaria. In both countries, there were periodic turnovers of leprosaria directors, sometimes recruited from outside the leprosaria. The appointment of leprosaria directors from other public hospitals and academic institutions, who had the intention to discharge the long-term residents, certainly could have opened a window.

Every time the issue reached the agenda, however, the proposed policy alternatives raised concerns among patients. Treating leprosy as an ordinary disease and integrating treatment into general medical care and public health measures threatened the 'special' status of leprosy. Many patients, who had become dependent on the leprosaria, feared losing their homes and privileges. If from a scientific perspective the disease no longer required isolation, patients sought to justify their continued privileges either as government duty to provide disease victims with compassionate care or as government compensation for past erroneous policies. Such patients' claims were not easily accepted. Given the lack of a public consensus on the moral underpinnings for patient support, the belief that long-term residents are entitled to generous government "compensation" was a hurdle. To surmount that hurdle, administrators and medical professionals would have had to acknowledge their wrongdoing and determine when the isolation policy could and should have been judged as obsolete. Additionally, public opinion, and of course the views of elected officials, would have needed to be nurtured to recognize and sympathize with patients' adversaries, allowing for generous government payments. As a result, the position of the patients was not really unified, and therefore they could not fully mobilize themselves politically. Consequently, this policy community more or less shared the view that the patients' disadvantages caused by the law were a necessary cost of its even greater benefits, namely the continuation of leprosaria[101].

When it proved impossible to accomplish decisive legislative changes, straightforward efforts to discharge long-term and medically unwarranted residents were also reduced. Instead policy adjustments were made by incremental administrative actions. Slow, limited and sometimes informal efforts were made for patients' discharge and for the provision of outpatient services. As one might expect[102,103], these disjointed and informal increments of change had limited success in modifying the policy's original effect. Since the policy under the existing law was administered on the premise that leprosy requires confinement and special care, it was limited in its capacity to compel patients to leave the leprosaria. As many patients chose to remain in their leprosaria, isolation ostensibly continued in the form of long-term residential care. In this way, the main focus of leprosy prevention policy gradually changed from social protection and patient care to the mere provision of residential places for patients and ex-patients (and later, rehabilitative services). These bureaucratic satisficing behaviors, as reported in other cases[104,105], could not raise again the fundamental question of law revision, and as a consequence many engaged themselves in pork barrel politics to preserve and improve life in the leprosaria. For many years, policy (change) focused on discrete, short-term outputs rather than broader, long-range outcomes[106].

In the 1980s, broad policy revision became possible again though the agendas were set by factors again other than science, specifically the discussion on economic inefficiency in the US and the issue of human rights violation in Japan. In the former, large government expenditure was a big social and political issue, and there was an active search for potentials for spending cuts. In the latter, AIDS as a source of social stigmas and the forced institutionalization of psychiatric patients were both prominent in the media, calling for the protection of the human rights of the diseased. Furthermore, as Bardach suggested[107], increased political competition and Cabinet turnover at that time may also have increased the likelihood that elected officials would take up potential social issues to be remedied. In the US, Representatives Baker and Field fought for a House seat, while in Japan the two major parties competed vigorously for government office.

An indispensable key to policy change was the development of policy alternatives. Interestingly, they were quite common in both countries. Evidently, policy change occurred only after the leprosaria were recognized as legitimate homes for some patients and outpatient services were officially established. Patients were assured of their residences, but had the option of leaving the institutions. The patient's privilege of remaining in a leprosarium was then justified as partial compensation for the past compulsory segregation. In both countries, hazard duty payments were also maintained for ongoing employees, who could have otherwise opposed to policy changes. The primary difference, as reflective of the key arguments against the old policy, i.e., economic inefficiency and human rights violation, was that in Japan a public apology was offered by the government for its long-term negligence, though the apology was later used by the patients to win further government compensation through lawsuits. Another point is that in the US, large stipends were awarded to patients as an economic incentive to reduce the number of institutional residents.

### The functions of science and scientists in the policy process

From the perspective of the Garbage Can model, problems, policies and politics typically evolve in separate, unconnected streams. When science is considered as a fourth factor, it is seen to influence all the streams, changing perception of problems, helping develop policies, and transforming political environments[108]. Thus, science can be seen as approving or disapproving certain (existing) policies. However, it does not automatically create those effects by itself, nor in a political vacuum. Even when science indicates that an existing policy is no longer relevant, as this case study shows, people may make objections to its abolition for a variety of other reasons. In comparison with basic research, furthermore, applied research, including public health and epidemiological research, is more likely to follow an agenda driven by forces other than science[109]. This means that the imminence of an issue, or its agenda status, can affect the production and use of scientific knowledge.

Furthermore, scientific disputes will not be always resolved during the time the scientific issues are considered relevant to policy discussions[110]. Looking back at Period I, Promin was first mentioned in Leprosy Review in 1945, but it was not until 1952 that sulfone therapy became accepted among academics[111,112]. Controversy on the effectiveness of sulfone drugs certainly still lingered at the time when the legislative changes were on the agenda, namely, around the time of the enactment of the Public Health Services Act and subsequent policy proposals in the US and at the time of the revision of Leprosy Prevention Law in Japan. The development and acceptance of scientific knowledge might have been slowed by the attitudes of authoritative and powerful experts in Japan. Also, in the US, quite a few experts continued to justify the isolation of infectious cases for many years. Arguments that did not support their convictions were sometimes screened out by disqualifying them. Authoritative positions of medical and administrative elites in society also hindered the public's critical appraisal of their arguments, as did the importance then attached to public health[113]. The strength of the conservatives' arguments possibly came also from the systemic bias in their position as defenders of status quo: they had to simply attack a proposer's case as insufficient[114].

In an adversarial process of rule-making, knowledge claims are sometimes deconstructed, exposing areas of weakness or uncertainty. These revealed weaknesses provide justification for political decision makers to assert that they have a right to engage in interpreting science, especially in areas that are controversial. Again, uncertainty within science itself is also a subject for negotiation, decision, and argument in policy process, since the quality and amount of knowledge are the objects of social negotiation[115]. This partial transfer of cognitive authority to the legal and political arena may be seen as the way of assuring that the interpretation of (indeterminate) facts reflects the public values embodied in the legislation as well as the norms of the scientific community[116]. The connections between given scientific data, expert interpretation of these data and policy content are like chains of linked arguments and beliefs[117]. This process might be termed co-evolution or the process of mutual validation between policy and science[118]. In a case where science and policy are co-constructed through processes which occur in tandem, it becomes difficult to explain the one by using the other[119]. From the vantage point of history, the very absence or scarcity of critical review among legislators and the public in reviewing scientists' statements on the risk of disease spread might reflect and indicate their own fear of the disease and indifference to patients' human rights. When scientific knowledge is used in policy development, scientists and/or policymakers may choose to err either on the side of public safety or on that of patients' liberty and dignity.

For a long portion of Period II, incomplete knowledge was not necessarily the primary reason why science did not play a major role. Scientists, experts and legislators could have acted strategically as policy entrepreneurs to take advantage of occasional opportunities, provided by several focusing events, to open the windows of opportunity. In other words, scientific opposition, or dissensus if any, was not a major hindrance to their actions. Scientists, however, preferred not to encroach upon policy debates in an official and overt fashion, thus avoiding direct questions about justification and continuation of the institutions. In both countries during the 1960s and 1970s, there was a near consensus, albeit implicit among the interested parties, including scientists, physicians, bureaucrats, patients and politicians, that the leprosaria should be preserved or at least not immediately abolished. Though scientific assessment appeared conclusive and potent enough if effectively presented, consideration for the possible negative consequences of policy outcomes constrained its use. In the resultant incremental decision making, the demand for science was minimal.

Later in period III, when development of policy alternatives was definitely the key to changing policies, the process was not fueled by (new) scientific knowledge or discussion, but rather propelled by political skills which crafted both the policies and the favorable environments. A scientific consensus had already existed before the issue was taken up at this point, which illustrates the secondary importance of science in the emergence of policy windows. At most, the known arguments were reiterated and pushed to the fore. Policy change was not really contested and disputed in terms of science, simply because it did not have to be once attractive policy alternatives were proposed.

From this vantage in all the periods, science remained instrumental in the sense that scientific rationality was not the primary objective for which policy change was discussed or intended, and in the sense that scientific arguments were not the principal driving force for policy change. To review the course of events: Some initiating event, not directly associated science, leads to a policy issue that must be decided on; a debate ensues on the possible policy options and on possible scientific views of the issue; a scientific assessment of the policy issue yields a rationale for a policy choice[120,121]. Thus, science is used either to legitimate policies developed for nonscientific reasons or is ignored if the consensus contradicts policy or there is scientific "dissensus." To the extent which science can be regarded as instrumental, namely that final policy decision was rendered to lawmakers, the role and responsibility of science could decrease[122]. The scientific base and the political will to translate it to policy are ingredients necessary to induce policy changes[123-125]. Inertia and/or political considerations certainly exerted huge influence[126].

### Policy entrepreneurs and leadership

Although many contextual conditions could facilitate changes in health policies, they do not in themselves activate actions by either legislators or administrators[127-129]. As is the case with policy adoption[130,131], successful termination might need a political leader, a terminator, to trigger and manage effectively the process of policy termination. He must cultivate support from both influential interest groups and from the general public and choose the arena for policy discussions[132-134]. Then, a person skilled in policy termination should get the termination on the agenda, orchestrate advocacy coalitions, negate the survival tactics of anti-termination coalitions, and manage the administrative details[135-138]. With respect to HD policy, for a long period, intellectual reluctance, the tendency to institutional permanence, dynamic conservatism, efficacious anti-termination coalitions, and legal obstacles certainly posed difficulties for policy changes in both countries[139,140]. After a set of these contextual conditions were met, however, entrepreneurs had to accomplish their tasks. A crucial step for policy termination, namely the translation of ideas into action by coupling concrete and acceptable policy proposals with problem situations and political opportunities, was accomplished by a policy termination expert[141,142].

When things proceed as the Garbage Can model suggests, policy change requires a good luck and/or a skillful policy entrepreneur, who can induce the convergence of streams and open the windows of opportunity. This study highlighted an essential role assumed by a skillful terminator, Baker in the US and Otani in Japan. In the US, Representative Baker, along with the PHS officials, held meetings with the patients and staffs at the institution so that all interested parties could reach agreement on his proposals. The PHS publicized its own review of the institution and its activities highlighting their economic inefficiency. In this way, Baker and the PHS maneuvered the political process to successful legislation enactment. In Japan, Otani dramatized the evils of the old policy, stressing the benefits of termination, while mollifying the worried patients by reassuring of their continued residence in the sanatoria. He thus successfully engineered a consensus among key interest groups, publicizing a thorough justification for termination and devising an acceptable plan. Through political leadership and skill, the issue was finally brought to the forefront, alternatives supplied, obstacles cleared, and the policy abolished. Both Baker and Otani conducted a series of bandwagon activities making effective use of the media.

## Conclusion

Quarantine measures aim to protect the health and safety of the public, and consequently individual liberty is subordinate to the common good[143]. Parens patriae, the obligation of the state to act as parent of the country in caring for those who cannot care for themselves, is the other side of the duty of the state to protect the rest of the community from infected individuals[144]. The human rights or civil liberties of patients must be weighed the effectiveness and efficacy of the isolation measure. It is inarguable that science could and should play a key part.

The utilization of scientific knowledge contributes to increased rationality of health policy, which is desirable especially when incongruence could produce adverse effects. However, when policy changes occur as a result of the convergence of three largely independent streams of problems, policies and politics, we cannot assume a rational and linear process which automatically incorporates science into policy. As discussed, a variety of dynamics influence the utilization of scientific research in policymaking[145-147]. For policies to be reflective of science, these models suggest, the interactions among researchers and policy makers are critical. Research is more likely to be utilized in a significant way when effective networks and mechanisms are established at the interfaces among researchers and policy makers[148]. However, even that may not be sufficient.

Events in the various streams should be made readily understandable and visible, either in hopes of a serendipitous convergence or so that policy entrepreneurs can assist in their convergence, in order to open the window of opportunity. Problems should be foregrounded, political factors should be elucidated, and feasible and attractive policy alternatives should be envisioned. Experts should be mobilized to assume authoritative roles in linking science and policy, though it should always be noted that they too could be biased in one way or another. Political empowerment of the people who suffer from the negative policy outcomes, and therefore might be most attentive to them, could also help serve the purpose.

Policy is always developed both from scientific and nonscientific considerations, as this study indicated. The Possibilities for the instrumental use and/or disuse of science should be made explicit to the public. Mobilization of different social views and values, in comprehensive consideration of direct and indirect policy impacts, could lead to a more explicit and formal social process, which could eventually secure and propel the process of muddling through[149]. Public governance over these processes, along with public's enlightenment and mobilization, might facilitate, check and complement the functions fulfilled by the experts. Analysis of policy and politics is the indispensable key to these processes.

## Competing interests

The author(s) declare that they have no competing interests.

## Authors' contributions

All the authors (HS, JF) fully participated in the planning of research, the collection of historical materials, and the carrying out of interviews in both the US and Japan. After thorough discussion, HS drafted the article, and both authors read and approved the final manuscript.

## Pre-publication history

The pre-publication history for this paper can be accessed here:


